# A survey comparison of educational interventions for teaching pneumatic otoscopy to medical students

**DOI:** 10.1186/s12909-019-1507-0

**Published:** 2019-03-12

**Authors:** Alanna Higgins Joyce, Maya Raman, Jennifer L. Beaumont, Heather Heiman, Mark Adler, Suzanne M. Schmidt

**Affiliations:** 1 Department of Pediatrics, Northwestern University Feinberg School of Medicine, 225 East Chicago, Box 86, Chicago, IL 60611 USA; 2 Department of Medical Social Sciences, Northwestern University Feinberg School of Medicine, 633 N. Saint Clair Street, 19th Floor, Chicago, IL 60611 USA; 3 Present address: Terasaki Research Institute, 1018 Westwood Blvd, Los Angeles, CA 90024 USA; 4 Department of Medicine, Northwestern University Feinberg School of Medicine, 51 East Huron Street, Galter Suite 3-150, Chicago, IL 60611 USA; 5 Ann & Robert H. Lurie Children’s Hospital of Chicago, 225 East Chicago Ave, Box 152, Chicago, IL 60611 USA

**Keywords:** Simulation, Otoscopy, Ambulatory medicine, Pediatrics, Medical student, Medical education research

## Abstract

**Background:**

Though pneumatic otoscopy improves accurate diagnosis of ear disease, trainees lack proficiency. We evaluated the effect of three different training techniques on medical students’ subsequent reported use of basic and pneumatic otoscopy in patient encounters.

**Methods:**

Pediatric clerkship students participated in an ear exam workshop with randomization to one of three educational interventions: task trainer (Life/form®, Fort Atkinson WI), instructional video, or peer practice. Each student received an insufflator bulb and logbook to record otoscopic exams and completed an 18-item anonymous survey at clerkship conclusion.

**Results:**

115 of 150 students (77%) completed the survey. There was no significant difference in number of basic or pneumatic otoscopic exams performed based on method of training. Most students (68–72%) felt more likely to perform pneumatic otoscopy after training. Though the majority of students performed basic otoscopy on patients when an ear exam was indicated, they used pneumatic otoscopy less than 10% of the time. Students reported significant barriers to otoscopy: time, access to equipment, cerumen impaction, patient hold, and anxiety. Student comments described a culture where insufflation was neither practiced nor valued by supervising physicians.

**Conclusion:**

Training in pneumatic otoscopy can increase student comfort, but barriers exist to using the skill in clinical practice.

## Background

Acute otitis media (AOM) is one of the most common reasons for pediatric medical consultation worldwide, accounting for up to 40% of medical visits within the first five years of life [1]*.* Nearly 80% of US children younger than two years of age are affected by AOM, with AOM spending totaling $2.8 billion in the year 2006 [2, 3]*.* In addition, AOM is the most common indication for antibacterial treatment of children in the US, making accurate diagnosis vital in the face of rising antibiotic resistance and health care costs [4, 5]*.*

Pneumatic otoscopy consists of the use of insufflation to assess mobility of the tympanic membrane (TM). The pneumatic component of otoscopy improves accurate determination of presence or absence of a middle ear effusion (MEE), an essential characteristic in the diagnosis of otitis media with effusion (OME) [6]*.* It also aids in determining TM position (bulging, retracted or neutral), and accurate diagnosis of AOM with a sensitivity > 90% and specificity of nearly 80% [4]. The American Academy of Pediatrics (AAP) 2013 Clinical Practice Guidelines state that clinicians “should not diagnose AOM in children who do not have MEE (based on pneumatic otoscopy and/or tympanometry).” [7] Multiple studies support that bulging of the tympanic membrane is positively correlated with recovery of bacterial pathogens from the middle ear fluid [8, 9]*.* In addition, guidelines recommend against the use of systemic antibiotics for the treatment of OME [10]*.* By using pneumatic otoscopy to more accurately diagnose AOM and OME, physicians may avoid unnecessary antibiotic treatment or further interventions [11]*.* Training in and utilization of pneumatic otoscopy and tympanometry by general practitioners have been shown to improve practitioner confidence [12] and increase inter-rater agreement in the diagnosis of ear disease, decreasing rates of AOM diagnosis by 30% [13]*.*

Despite expert recommendations, physicians and trainees do not often use or teach pneumatic otoscopy. A study of primary care physicians showed that while 90% of physicians had read the AOM guidelines, more than half of physicians surveyed did not use pneumatic otoscopy regularly [14]*.* In another report, pneumatic otoscopy was considered an advanced technique, with many physicians unconvinced that the additional training and effort would result in patient benefit, and two physicians declined to use the skill during the study [15]*.* Identified barriers to teaching otoscopy include educator discomfort with teaching the skill and lack of equipment availability [16]*.* One study of Italian pediatricians and otolaryngologists revealed that only 9% of these physicians were taught to diagnose and treat AOM in medical school; however the more education about AOM these physicians received, the more positively they viewed AOM guidelines [17]*.* The AAP encourages instruction in evaluation of the middle ear beginning in medical school, with continuing medical education to “reinforce the importance of, and retrain the clinician in, the use of pneumatic otoscopy” [7]. Additional teaching may improve attitudes towards and adherence to AOM guidelines.

Existing educational methods for otoscopy include reading or watching videos, with some use of simulation and standardized patients. However, medical educators are heeding the call to enhance education through active learning [18]. Experience performing clinical skills should raise the comfort level of the learner, increasing their likelihood of putting the skill into practice. Simulation-based education has been shown to improve learner outcomes,[19] although it also may require more resources than traditional curricula [20]. Thus, determining the impact of different educational methods on use of pneumatic otoscopy is important to ensure development of efficient and effective curricula to teach this essential skill.

Previous studies have shown that brief training sessions in simple and pneumatic otoscopy can improve medical student, resident, and physician confidence and accuracy [12, 21, 22]. In one single-center study, training with an ear exam simulator, the “Life/form® Diagnostic & Procedural Ear trainer with Pneumatic Otoscopy Kit,” improved the ability of medical students to apply appropriate pneumatic pressure during insufflation and to identify the presence of a middle ear effusion with 100% accuracy [23]. No study has yet evaluated the effect of simulator training on trainees’ subsequent use of basic and pneumatic otoscopy on real patients. Though it is clear that better training is needed in pneumatic otoscopy, the optimal method of delivering this content has not been identified.

## Aim

Our objective was to evaluate the effect of three different training techniques in pneumatic otoscopy on medical students’ subsequent use of basic and pneumatic otoscopy in patient encounters during the third year pediatric clerkship. We hypothesize that training using an ear exam simulator with real-time feedback will yield increased medical student performance of this skill, when compared to traditional educational methods.

## Methods

Subjects included third year medical students on their core pediatric clerkship at Northwestern University’s Feinberg School of Medicine. The pediatric clerkship is six weeks in duration and provides experience with hospitalized patients, community ambulatory pediatrics, and brief time in the newborn nursery, urgent care, and neonatal intensive care unit. Prior to entering clerkships, all students have instruction in and practice with basic and pneumatic otoscopy, and are assessed on their physical exam skills including simple otoscopy. All medical students on the pediatric clerkship during the 12-month study period spanning a full academic year were eligible for the study. Approval was obtained from the Feinberg School of Medicine and the Institutional Review Board.

**Fig. 1:**
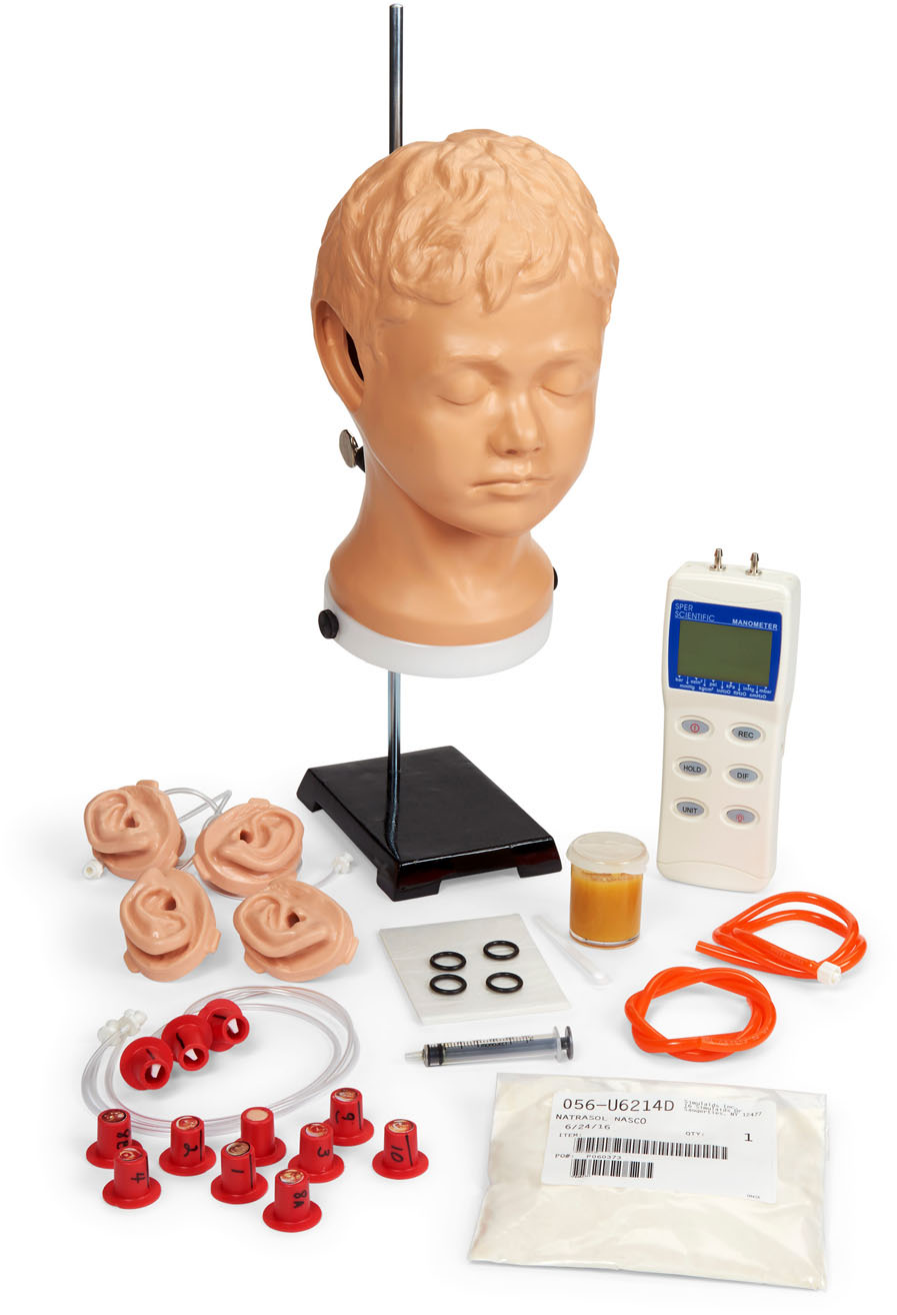
Task trainer: Life/form® Diagnostic & Procedural Ear Trainer with Pneumatic Otoscopy Kit *(reproduced with permission from vendor* *www.enasco.com**)*

Medical students participated in a 90-min workshop on advanced otoscopy skills during the first two days of their pediatric clerkship. The workshop consisted of a standardized 30-min didactic presentation followed by an educational intervention. Students seated in alphabetical order “counted off” by threes for assignment to one of three different educational intervention groups: (1) training on a task trainer (Life/form®, Fort Atkinson WI), (2) independently watching an instructional video in a small group (New England Journal Videos in Clinical Medicine: Diagnosing Otitis Media - Otoscopy and Cerumen Removal), and (3) peer-to-peer practice on each other using otoscopes with insufflator bulbs. The task trainer, “Life/form® Diagnostic & Procedural Ear Trainer with Pneumatic Otoscopy Kit,” was created by a collaborative group of undergraduate engineering students, medical students, residents, and faculty at the University of Virginia [23]. The task trainer consists of a mounted pediatric mannequin head with a soft, detachable ear that has a simulated mobile tympanic membrane (Fig. 1). The model can be configured to measure insufflation pressure on an attached manometer during pneumatic otoscopy.

Training on the task trainer began with direct instruction by the clerkship director on use of the simulator, followed by individual student practice on the task trainer with goals of obtaining an adequate seal with the speculum and using appropriate insufflation pressure, as measured by the manometer. Peer-to-peer practice was student-led practice of otoscopy and pneumatic otoscopy with the goal of visualizing peers’ tympanic membranes and TM mobility. Video participants viewed the module as a group in an adjacent room. The clerkship director circulated among all three groups and was available for questions throughout the interventions, which occurred simultaneously.

On the first week of the rotation, each student was given an insufflator bulb for use throughout the clerkship and a log book to record all otoscopic exams performed throughout the six-week clerkship. The task trainer was available to all students for optional practice halfway through the clerkship. At the conclusion of the clerkship, students were asked to complete an 18-item web-based anonymous survey reporting their experiences with free-text feedback comments allowed. Students gave consent by completing the survey. Survey responses did not contain any student identifiers and the survey was not required.

Survey results were analyzed and free-text feedback reviewed. Groups were compared using chi-square tests for categorical variables and non-parametric Kruskal-Wallis tests for continuous variables. A sample size of 38 per group allowed for the detection of moderately sized differences of 0.66 SD units, at 80% power and two-sided alpha of 0.05.

## Results

Of 150 clerkship participants, 115 completed the survey, of whom 39 students were randomized to the task trainer, 37 to video instruction, and 38 to peer-to-peer practice. Roughly half of the participants were female and had completed 1–2 clerkships (Table 1). Nearly three-quarters reported no prior experience with pneumatic otoscopy.

**Table 1:** Student demographics and clinical experience

	Task Trainer (*n* = 39)	Video Presentation (*n* = 37)	Peer-to-Peer (*n* = 37)
Gender			
Female	18 (46%)	15 (41%)	19 (51%)
Male	21 (54%)	22 (59%)	18 (49%)
Number of Prior Clerkships Completed			
1–2 clerkships	18 (46%)	19 (51%)	15 (41%)
3–4 clerkships	10 (26%)	8 (22%)	13 (35%)
≥ 5 clerkships	11 (28%)	10 (27%)	10 (27%)
Prior Experience with Pneumatic Otoscopy			
None	28 (72%)	28 (76%)	25 (68%)
1–5 times	11 (28%)	8 (22%)	10 (27%)
> 5 times	0 (0%)	1 (3%)	3 (8%)

Sixteen percent of students reported using the task trainer at the halfway point of the clerkship (13% of the task-trainer group, 19% of the video instruction group and 16% of the peer-to-peer practice group; chi-square *p* = 0.767).

The median number of basic otoscopic exams performed during the six-week clerkship was 70 for the task trainer group (range 22–300), 62 for the peer-peer practice group (range 16–186), and 60 for the video instruction group (range 20–200). The median number of pneumatic otoscopic exams performed was 4 for the task trainer group (range 0–80), 4 for the peer-peer practice group (range 0–70), and 2 for the video instruction group (range 0–50) (Table 2). Using an Independent Samples Kruskal-Wallis, the difference among the groups was not significant for either outcome (*p* = 0.455, *p* = 0.253).

**Table 2:** Number of basic and pneumatic otoscopy exams performed during clerkship

	Number of Basic Otoscopy Exams Performed		
	Median	IQR	Range
Task Trainer	70	40–100	22–300
Peer-to-Peer	62	50–100	16–186
Video Instruction	60	40–80	20–200
	Number of Pneumatic Otoscopy Exams Performed		
	Median	IQR	Range
Task Trainer	4	1–12	0–80
Peer-to-Peer	4	0–10	0–70
Video Instruction	2	0–6	0–50

**Fig. 2:**
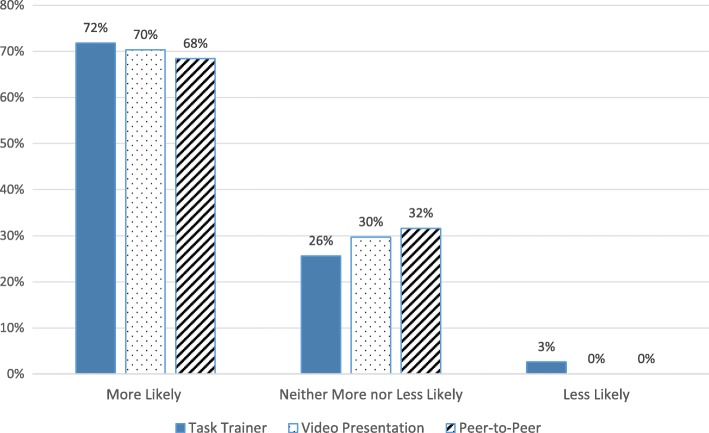
Student likelihood to perform otoscopy following training

**Fig. 3:**
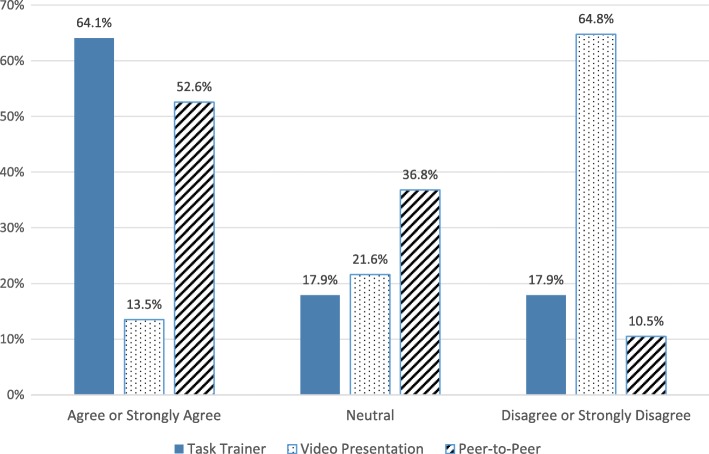
Effectiveness of educational interventions in preparing students to perform pneumatic otoscopy

**Fig. 4:**
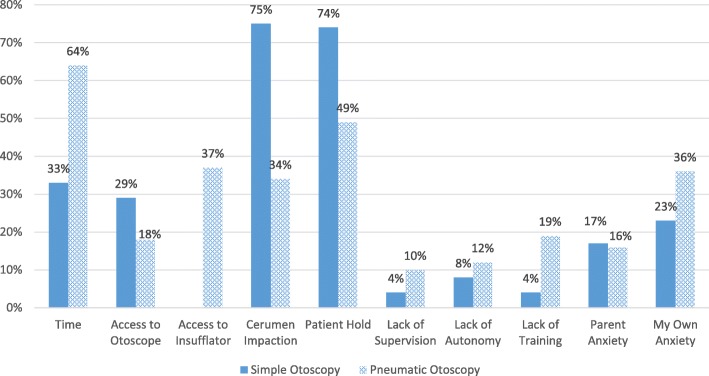
Student-reported barriers to simple and pneumatic otoscopy

The majority of students in all groups (68–72%) indicated they were more likely to perform otoscopy after their training (Fig. 2). Students who trained on the task trainer or with peer-to-peer practice agreed or strongly agreed that their training adequately prepared them to perform pneumatic otoscopy on patients (64 and 53% respectively), compared to 14% of those who watched the video (Fig. 3). The vast majority of students (75%) reported performing basic otoscopic exams on more than 80% of patients younger than 5 years of age who needed an ear exam. However, most students (68%) said they used pneumatic otoscopy in less than 10% of their ear exams (62% task trainer, 71% peer-to-peer, 70% video; *p* = 0.612). Major barriers to basic and pneumatic otoscopy included cerumen impaction, time, anxiety, and patient hold (Fig. 4).

Student feedback results described a culture where pneumatic otoscopy was neither practiced nor valued by multiple supervising physicians during their rotations (Table 3). One student noted “no attendings used insufflation in practice, and I think this was the biggest barrier to using the insufflator… when I was in a room alone, I was more likely to use the insufflator.” Students noted that when preceptors did not use insufflation, it felt “unnecessary” and “awkward” for the student to do so. One student commented that the “outpatient office asked [them] not to do it” and another said they were told it “hurt patients.”

**Table 3:** Selected student comments on barriers to performing pneumatic otoscopy

Group	Comment
Task Trainer	I did not see ANY other providers doing pneumatic otoscopy. Not a single attending or resident. This sort of made it feel like an added part of the exam that was not quite necessary.
	[Pneumatic otoscopy] was not used at all by supervising physicians and was regarded as unnecessary.
Video Presentation	No attendings used insufflators in practice, and I think this was the biggest barrier to using the insufflator, myself. I found that when I was in a room alone, I was more likely to use the insufflator.
	Outpatient office asked me not to do [pneumatic otoscopy].
Peer-to-peer	Preceptor advised [me] not to use pneumatic exams because they hurt patients.
	I didn’t see one attending use an insufflator, and I followed suit at first just to save time, but then got out of the habit and stopped.

## Discussion

Given the importance of proficiency in basic and pneumatic otoscopy in pediatrics, as well as the frequent lack of formal training, we evaluated three different educational interventions to teach these skills to third year medical students in order to determine optimal educational methods of teaching this skill. Though all groups reported improved comfort with and likelihood of performing pneumatic otoscopy after the educational intervention, no single intervention was associated with a greater subsequent use of basic or pneumatic otoscopy in the clinical setting, assessed by number of exams performed by each student. Previous studies of pneumatic otoscopy simulation assess learners’ subsequent skill in simulated settings [24]. To our knowledge, this is the first controlled study comparing simulator training with other learning techniques, and the first to examine the outcome of subsequent use of basic and pneumatic otoscopy in the clinical setting.

Students assigned to a “hands-on” intervention (peer-to-peer practice or the task-trainer) felt more prepared to perform the pneumatic otoscopic exam in the clinical setting than those who watched the video. These findings suggest that incorporation of one of these active learning techniques is important and can help guide development of optimal training in otoscopy skills at multiple levels. In our study, learning with the task trainer was not shown to be superior to peer-to-peer practice on a fellow student in improving comfort with the exam or in the number of exams performed in the clinical setting. Reasons for this could include limitations of the model itself or its utilization. Using additional features of the model, such as instilling fluid into the middle ear of the model or showing examples of different ear findings could affect the results. Our results could have been improved by additional practice with the task trainer at the mid-clerkship point facilitated by an expert or an activity with attached academic credit.

Most students reported performing basic otoscopy on “patients under five who needed an ear exam.” Parameters were left to provider discretion, as patients were seen diverse clinical settings such that an ear exam was not always warranted. In addition, the majority of students in all groups indicated they were more likely to perform pneumatic otoscopy after their training than before. Despite improved attitudes, 68 % of students reported performing pneumatic otoscopy in less than 10% of their ear exams. This suggests that additional work is needed to overcome the disconnect between student comfort with a clinical skill and the application of that skill in a clinical setting.

Students cited multiple barriers to the use of insufflation on pediatric patients (Fig. 4), and student feedback described a culture where insufflation was neither practiced nor valued during their rotation (Table 3). While we were able to address some of the barriers cited in previous studies, including equipment availability and formal training, we encountered other unexpected obstacles including the lack of faculty performance of pneumatic otoscopy, and in some cases resistance to it. Further research is underway to explore preceptors’ barriers to teaching and using pneumatic otoscopy.

Our study has several additional limitations. As a single-institution study, findings may be specific to our school, community practice patterns, or educational priorities. Our outcome measures were based on student report, which is subject to recall and social desirability bias, in which students may over report an outcome to please the surveyor. Less engaged students who did not respond to the survey may also be the least likely to perform these clinical maneuvers. Finally, we lack performance data or evaluation of the students’ otoscopy skills in the clinical setting, which would better elucidate the effectiveness of our interventions.

With continued study of students and preceptors at multiple centers, we hope to better delineate barriers to successful performance of pneumatic otoscopy and strategies to overcome them. Understanding the perspective of clinical preceptors will be essential to addressing obstacles to teaching students pneumatic otoscopy. Our results can help guide the creation of interventions that target the continuum of learners, from medical students to residents to clinical preceptors. The development of strategic and consistent medical education, coupled with continued training for front-line practitioners and educators has the potential to increase the appropriate use of advanced otoscopy skills and improve accurate diagnosis of ear disease.

## Conclusions

We evaluated three different educational interventions to teach basic and pneumatic otoscopy to third year medical students: task trainer (Life/form®, Fort Atkinson WI), instructional video, and peer practice. Training with any of these methods increased student comfort with otoscopy, but did not result in increased use of basic or pneumatic otoscopy in the clinical setting. We identified multiple barriers that deter students from putting into practice the skills they have learned during patient encounters. Continued study of students and preceptors in the clinical setting is needed to explore barriers to successful performance of pneumatic otoscopy and to identify strategies to overcome them.

## Abbreviations

AAP: American Academy of Pediatrics
AOM: Acute Otitis Media
MEE: Middle ear effusion
OME: Otitis media with effusion
